# Cognitive reserve modulates mental health in adulthood

**DOI:** 10.1007/s40520-024-02776-w

**Published:** 2024-07-02

**Authors:** Daniele Porricelli, Margherita Tecilla, Veronica Pucci, Elisa Di Rosa, Sara Mondini, Marinella Cappelletti

**Affiliations:** 1 Psychology Department, Goldsmiths University of London, London, UK; 2 Department of General Psychology, University of Padua, Padua, Italy; 3 Department of Philosophy, Sociology, Education and Applied Psychology, University of Padua, Padua, Italy; 4 Human Inspired Technology Centre (HIT), University of Padua, Padua, Italy; 5 Department of Developmental Psychology and Socialization (DPSS), University of Padua, Padua, Italy; 6 IRCCS San Camillo Hospital, Venice, Italy

**Keywords:** Cognitive reserve, Mental health, Middle age, Ageing

## Abstract

Cognitive Reserve (CR) reflects acquired knowledge, skills, and abilities throughout life, and it is known for modulating cognitive efficiency in healthy and clinical populations. CR, which was initially proposed to explain individual differences in the clinical presentation of dementia, has subsequently been extended to healthy ageing, showing its role in cognitive efficiency also during middle age. Recently, CR has been linked to affective processes in psychiatric conditions such as schizophrenia, major depressive and anxiety symptoms, and psychological distress, suggesting its potential role in emotional expression and regulation. Whether the role of CR in mental health extends to non-pathological adults, and whether this is only relevant in older age is not yet clear. The aim of this work was therefore to explore the relationship between CR and mental health in healthy adults, with a focus on middle adulthood (40–60). In a sample of 96 participants, we found a positive association between CR and mental health outcomes, such that a higher cognitive reserve index corresponded to fewer mental health reported symptoms. Specifically, a higher CR reflecting professional activities was associated with lower stress levels, especially in middle agers. Taken together, these data therefore suggest that engaging occupations may help maintain a robust mental health, especially by reducing stress symptoms during middle age. These results broaden previous findings suggesting that CR relates to affective components of mental health in middle aged and older adults.

## Introduction

Cognitive Reserve (CR) refers to knowledge, abilities and skills acquired during the lifespan, and may account for the adaptability of cognitive processes to pathology or insult, and to age-related changes [1]. CR mirrors ‘brain reserve’, which reflects the neuroanatomical resources underlying individual differences in brain structure such as the density of neuronal synapses, and cognitive efficiency like processing speed [2]. Both cognitive and brain reserves show high individual variability, with some adults more successful than others in maintaining the structural and functional properties of their ageing brains [2]. A common observation is that adults with higher CR typically have more efficient brain networks that allow them to actively cope with pathology using compensatory mechanisms [3–6], including at advanced age [3, 7]. This ‘compensation’ has been explained in terms of an accumulation of mental resources related to prolonged exposure to cognitively stimulating activities which can be used to mitigate the effects of physiological ageing [8].

CR has been operationalized as a composite measure of different indicators reflecting life experience in different areas, which contribute to building CR, specifically at school, work, and during free time [9]. Indeed, using experience-based CR indicators offers a more accurate, reliable, and complex representation of CR than referring only to sociodemographic variables such as educational attainment or verbal intelligence [10–12]. Formal education is indeed considered a static proxy reflecting a specific time frame in early life [13], which can reliably predict healthy ageing only in case of a correlation with the type of work and intellectual lifestyle [14, 15]. Therefore, a composite and dynamic measure of CR rather than a unique and static index (years of education) provides better construct validity for measuring CR [16].

The idea of CR has been initially formulated to explain the variability of symptoms in patients with dementia [17], with numerous studies in the context of the most common neurodegenerative disorder, i.e. Alzheimer’s disease [18]. CR has been studied in other neurodegenerative conditions such as Parkinson’s disease (e.g., [19–22]) and multiple sclerosis [23, 24], as well as in relation to the cognitive outcome of traumatic and acquired brain injury [25–27] and stroke [28, 29]. CR has also been applied to the study of healthy ageing, specifically age-related changes in cognition. For instance, CR fosters cognitive efficiency not only during later adulthood (over 60), but also during middle-ageing, i.e. between the age of 40 and 60 years old [28, 30–33]. Although significant age-related changes in brain structure and in cognition are present in old age, such changes begin to emerge earlier in adulthood [31, 34, 35], thus suggesting that in middle age, CR may already buffer the effect of cortical thinning on cognitive abilities such as memory or executive functions (e.g. [36]) with protective and compensatory actions.

Recently, CR has been related to the cognitive outcome of psychiatric and affective disorders, such as schizophrenia [37–40]), depression [41, 42], anxiety disorders [43] and COVID-19 [44]. Indeed, although CR has long been studied in relation to cognitive performance, recent evidence suggests that CR could also contribute to emotion expression and regulation, which play a significant role in the development of psychopathology [38, 40, 45, 46]. However, this evidence is limited to the study of pathological conditions and does not yet extend to investigations on affective functioning among healthy individuals.

Capitalising on these premises, the present study aimed at examining the link between CR and mental health cross-sectionally in a sample of healthy adults from the age of 40 to 75. By strategically focusing on a large age range, we aimed to first test a possible relationship between CR and mental health in healthy adults, and secondly to examine at what point in adulthood any such link may emerge. Indeed, some clinical conditions such as depression, anxiety, and stress-related disorders can often be amplified during middle age (40–60). For example, a recent epidemiological study showed that the peak of anxiety disorders onset appears to be during middle age, with a decrease in older ages [47]. Furthermore, Li and colleagues recently showed that in a large cohort of almost 16,000 individuals, depression was higher in middle age than in older people [48].

## Materials and methods

### Participants

A total of 96 healthy adults provided written consent and received monetary compensation to participate in this study which was approved by the local Ethics Committee at Goldsmiths, University of London. None of the participants had a history of neurological or psychiatric disorders, was under regular medication, or showed major cognitive impairments assessed with the Mini-Mental State Examination (MMSE [49]; only for participants over 60 years). The sample, with a mean age of 56.5 ± 11.8, was divided into two age groups: Middle Age (mean age 48.1 ± 6; age range 40–59; 57 participants; 30 females), and Older (mean age 68.7 ± 6 69.3 ± 5.5; age range 60–80; 39 participants; 36 females).

### Measures

All participants were administered: (1) The Cognitive Reserve Index questionnaire (CRIq [9], to measure their CR; and (2) A mental health self-reported questionnaire, the short version of the Depression Anxiety Stress Scale (DASS-21) [50, 51].

*Cognitive reserve index questionnaire (CRIq).*

The CRIq includes demographic data (e.g. age, gender, and marital status), and 20 items grouped into three sections referring to education (CRI-Education), working activity (CRI-WorkingActivity), and leisure time (CRI-LeisureTime), each with a specific sub-score [9]. CRI-Education includes years of education, in addition to any training carried out during the lifespan. CRI-WorkingActivity index reflects data on professions carried out during adult life, with a duration expressed in years. Based on the cognitive demands and responsibilities of a job, there are five distinct occupational levels available: unskilled manual work, skilled manual work, skilled non-manual or technical work, professional occupation, and highly intellectual occupation. CRI-LeisureTime covers cognitively stimulating activities carried out during leisure time and including intellectual, social and physical activities. The CRI total (CRIq score) is the average of the three subscores. The higher the CRIq scores, the higher the estimated CR. To account for the age effect, these CRIq scores were calculated with linear regression models with age as the independent variable, and row scores as dependent variables. The residuals from these regression models were standardised and adjusted to a scale with a mean of 100 and a standard deviation of 15. CRI could be classified into five ordered levels: Low (less than 70), Medium–low (70–84), Medium (85–114), Medium–high (115–130) and High (more than 130).

#### Depression, anxiety, and stress scale (DASS)

The Depression Anxiety Stress Scales – Short Version (DASS-21) [50, 51] is a 21-item scale for the assessment of subjectively perceived symptoms of Depression, Anxiety, and Stress. For each question, a 4- point Likert scale (score 0–3) resulted in three independent sub-scale scores for each mental health condition, ranging from 0 to 42 (21 × 2), adding to a total sc​​ore from 0 to 126. Lower total and individual subscores corresponded to better mental health.

## Data analysis

Descriptive statistics and variables’ distributions are reported in Table 1. Since the variables were not normally distributed, separate Spearman's ρ correlations investigated the relationship between Age, Cognitive reserve Index (CRI), and Mental Health (DASS) in the whole sample.

**Table 1:** Descriptive statistics of the whole sample and of the two sub-groups (Middle Agers and Older)

	Mean, (SD)			Range		
	Global	Middle Age	Older	Global	Middle Age	Older
Age	55.6 (11)	48.2 (6)	68.7 (6)	40–80	40–59	60–80
CRI- Total	118.5 (16.8)	112 (13.8)	128.3 (16.4)	85–162	85–162	90–160
CRI-Education	119 (13)	114.2 (10.1)	126.2 (13.7)	91–152	91–141	91–152
CRI-Work	108.9 (19.3)	104.6 (14.9)	115.3 (23.2)	71–162	80–147	71–162
CRI-Leisure	114.8 (19.5)	108.9 (15.6)	123.7 (21.5)	76–179	76–157	85–179
DASS Total	8.7 (7.7)	9.9 (7.7)	6.8 (7.4)	0–37	0–37	0–29
DASS Stress	4.1 (3.7)	5 (3.5)	2.8 (3.5)	0–15	0–14	0–15
DASS Anxiety	1.5 (2)	1.7 (2.3)	1 (1.3)	0–15	0–15	0–4
DASS Depression	3.1 (3.7)	3.2 (3.7)	3 (3.9)	0–16	0–16	0–16

Middle Agers (40–59 years old): *N* = 57; Older (60–80 years old): *N* = 39. Means and standard deviations in brackets. The CRI is classified into five levels: Low (< 70), Medium–low (70–84), Medium (85–114), Medium–high (115–130) and High (> 130). Higher DASS scores corresponded to more mental health reported symptoms (DASS range: 0–126; DASS subscales range: 0–42)

*CRI* Cognitive Reserve Index [9]; *DASS* Depression Anxiety Stress Scale (DASS-21; Short Version) [50, 51]

Separate, multiple linear regression models were performed, with DASS and its subscales (DASS-Stress, DASS-Anxiety and DASS-Depression) as dependent variables and CRI and its subscales (CRI-Education, CRI-WorkingActivity and CRI-LeisureTime) as as independent variables. Potential non-linear relationships between independent and dependent variables were also checked by introducing non-linear terms of the same variables within the models. In subsequent analyses, the same models were carried out separately in the two age groups (Middle-Age and Older). P-values were adjusted for False Discovery Rate.

## Results

### Between-group differences in CRI and DASS-21 scores

**Fig. 1:**
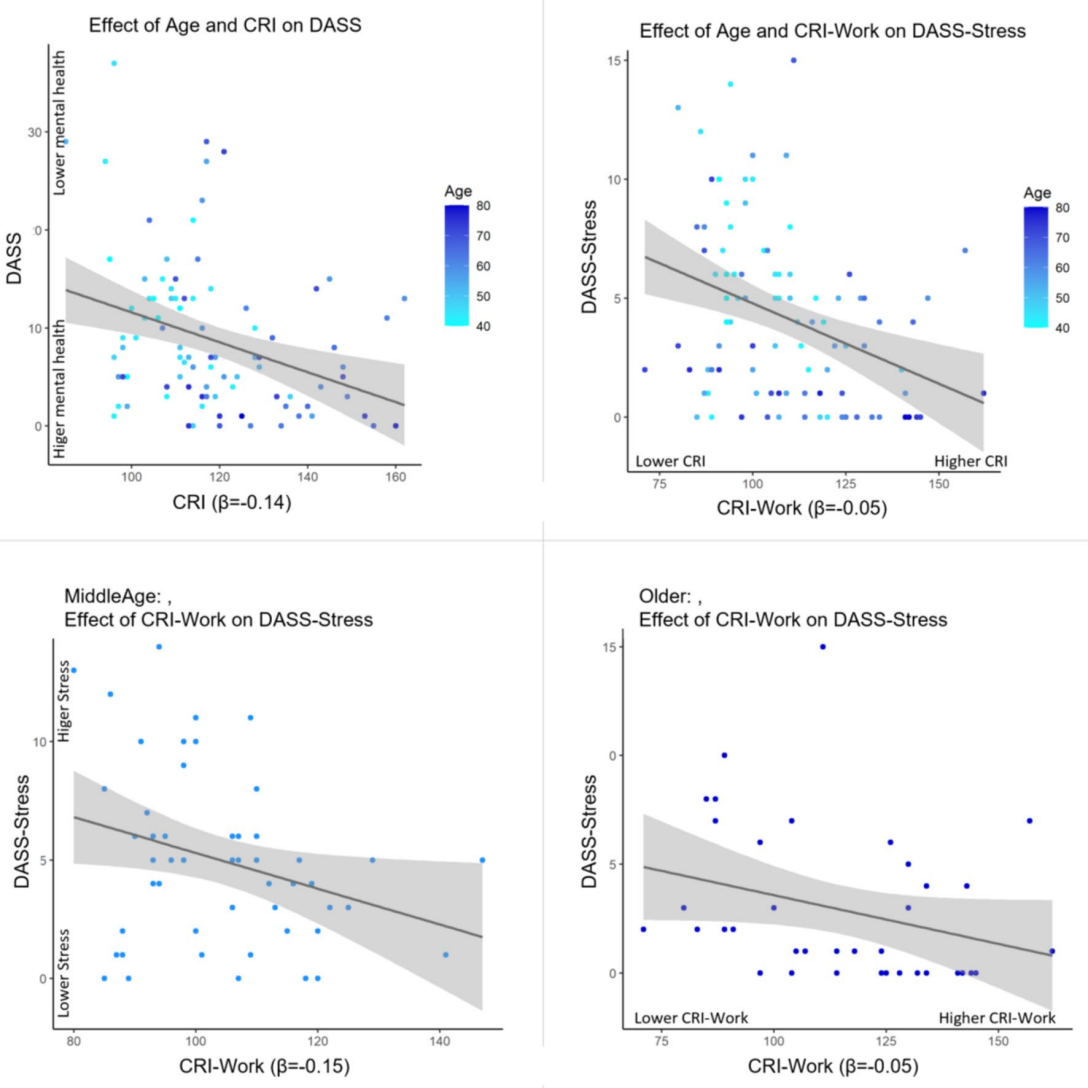
Link between Cognitive Reserve, Age, and DASS Age (40 to 80 years old) and Cognitive Reserve (CRI-Total index^1^) both significantly predicted mental health reported symptoms (total score of DASS^2^) across the entire sample (top left panel). Specifically, lower DASS scores indicating lower depression, anxiety and stress reported symptoms, emerged in older participants as well in participants with higher CRI levels, reflecting a greater estimated Cognitive Reserve. Age and Cognitive Reserve. The CRI-Working Activity index (indicating a higher Cognitive Reserve related to occupational activities), significantly predicted the DASS Stress subscale scores across the whole sample, such that lower DASS-Stress scores corresponded to higher age and higher CRI (top right panel). Strikingly, this association was only significant in middle-agers (bottom left panel) but not in older adults (bottom right panel). Beta values are reported in all panels.^1^CRI: Cognitive Reserve Index [9].^2^DASS, Depression Anxiety Stress Scale [50, 51]

The whole sample’s mean CRIq score was 118, with a significant difference between the two age groups (*t*(93) = −5.21,* p* < 0.001) because older adults showed higher CRIq scores compared to Middle-aged (see Fig. 1).

The mean total score of DASS was 8.7 (SD = 7.7), with middle agers reporting a higher number of mental health symptoms (mean = 9.9, SD = 7.7) than older participants (mean = 6.8, SD = 7.4). Although this difference was not statistically significant, there is a clear tendency towards a significance (*t*(94) = 1.97, p = 0.051), in accordance with previous research [52].

A significant difference between the two groups emerged when comparing the DASS-Stress scores (*t*(93) = 3.04, *p* = 0.003), with Middle-aged showing a higher score (i.e. higher amount of reported stress symptoms) relative to older participants (respectively 5 for middle agers and 2.8 for older adults). No difference emerged in the other subscales (see Table 1).

### Relationship between CRI and DASS-21 scores across the lifespan

A first correlation analysis based on the whole sample indicated that DASS-total score negatively correlated with the CRI score (*ρ* = −0.36, *p* < 0.001), such as higher CR levels were associated with lower symptoms of depression, anxiety and stress, and therefore with better mental health.

An equivalent analysis yielded significant correlations between Age and DASS total score (*ρ* = −0.30, *p* = 0.002), as well as Age and CRI (*ρ* = 0.54, *p* < 0.001), such as higher age was associated with both better mental health and higher CR levels.

### Role of the independent CRIq components and DASS-21 scores across the lifespan

Results of the regression analysis confirmed those based on the correlation approach, indicating that Age and CRI significantly predicted DASS total score, accounting for 11.5% (*R*^2^ = 0.115) of the variance across the entire sample (*F*(92, 2) = 5.98, *p* = 0.003). When analysing the contribution of the individual predictors in the model, we found that CRI was a significant predictor of DASS scores (βCRI = −0.14, *t*(92) = −2.61, *p* = 0.011). Specifically, lower DASS scores (i.e. lower depression, anxiety and stress reported symptoms) emerged in older participants, as well as in participants with higher CRI levels. Although Age and CRI correlated significantly, no multicollinearity arose in the regression analyses (Spermann’s *ρ* = 0.54), indicating that the two variables independently contributed to the models.

Additional models were conducted for the independent CRIq components, with results showing that only CRI-WorkingActivity had a significant effect on DASS total score. Age and CRI-WorkingActivity significantly predicted DASS total score, accounting for 10.5% (*R*^2^ = 0.105) of the variance across the entire sample (*F*(92, 2) = 6.49, *p* = 0.002). Likewise, DASS scores were also significantly predicted by CRI-WorkingActivity (βCRI-WorkingActivity = −0.11, *t*(92) = −2.79, *p* = 0.006), whereas CRI-Education and CRI-LeisureTime did not (respectively: *F*(92, 2) = 3.03, *p* = 0.278, and *F*(92, 2) = 3.24, *p* = 0.210).

### Role of the independent DASS-21 sub-scores and CRIq across the lifespan

Subsequent regression models with the three separate DASS sub scores as dependent variables indicated that CRI and Age significantly predicted only the scores of the DASS Stress subscale, such as lower DASS-Stress scores corresponded to higher age and higher CRI. In particular, Age and CRI significantly predicted DASS stress subscale, accounting for 17.1% (*R*^2^ = 0.171) of the variance across the entire sample (*F*(92, 2) = 9.43, *p* < 0.001). An analysis looking at the contribution of the individual predictors in the model showed that age and CRI were significant independent predictors (respectively: βage = −0.07, *t*(92) = −2.13, *p* = 0.036 and βCRI = −0.05, *t*(92) = −2.19, *p* = 0.031).

Strikingly, the CRI subscale with the strongest effect on the DASS-Stress scale was the CRI-Working Activity. In particular, Age and CRI significantly predicted DASS stress subscale accounting for 19.4% (*R*^2^ = 0.194) of the variance across the entire sample (*F*(92, 2) = 10.98, *p* < 0.001). Both age and CRI-WorkingActivity were significant independent predictors (βage = −0.08, *t*(92) = −2.76, *p* = 0.006, and βCRI-WorkingActivity = −0.05, *t*(92) = −2.74, *p = 0.007).*

### Role of DASS-21 sub scores and CRIq components in older and middle age adults

Given the significant effect of age in the total sample, separate linear regression models for Middle-age and Older groups were subsequently run to investigate potential age-related differences. Results of these models revealed that while CRI total score significantly predicted DASS total score in both groups (Middle Age: *F*(48, 1) = 3.06, *R*^2^ = 0.066; βCRI = −0.145, *t*(48) = −1.75, *p* = 0.05; Older Adults: *F*(36, 1) = 4.11, *R*^2^ = 0.103; βCRI = −0.144, *t*(48) = −2.03;,* p* = 0.049), CRI-WorkingActivity significantly predicted DASS-Stress scores only in the Middle-age group (Middle Age: *F*(48, 1) = 4.81, *R*^2^ = 0.091; βCRI-WorkingActivity = −0.07, *t*(48) = −2.19, *p* = 0.033; Older Adults: *F*(36, 1) = 3.43, *R*^2^ = 0.087; βCRI-WorkingActivity = −0.045, *t*(48) = −1.85;* p* = 0.07; see Fig. 1).

## Discussion

This study investigated the possible relationship between cognitive reserve (CR) and mental health in a sample of 96 healthy adults over the age of 40, considered as a whole, and separately in middle agers (40–59 years old) and older adults (60–80 years old). CR was measured using the CRIq [9], while mental health was evaluated using the DASS-21, which provides a self-assessed measure of depressive, anxiety and stress symptoms [50, 51].

Our results first showed group differences in both CR and mental health measures. Specifically, compared to middle agers, older adults had higher CRIq scores and lower stress symptoms. This finding was further supported by significant correlations between age and CR, as well as age and DASS scores. This is consistent with previous studies showing higher CR with advancing age, and age-related differences in stress level (e.g. [7, 53, 54]). The age difference in CR score also reflects the intrinsic nature of the working and leisure activities measured, since time spent on them tends to increase with age.

A second set of results indicated a significant relation in the entire sample between CR and mental health. Specifically, higher levels of overall CR corresponded to better mental health, as a compound index of depression, anxiety and stress symptoms. Next, to understand the nature of this relation, we specifically looked at the link between the distinct subscales in both the CRIq and DASS-21 in the two age groups independently. This showed that the association between CR and mental health was more strongly driven by a significant link between the DASS-stress subscale and the CR working activities in the middle age group only. Hence, higher levels of CR working activities predicted lower levels of stress in middle aged healthy adults.

These findings suggest that while CR may be a protective factor for mental health in the healthy population, it seems to have a stronger influence in middle agers because of cognitively stimulating working activities that might reduce stress symptoms.

Taken together, these data therefore suggest that engaging occupations may help maintain a robust mental health, especially by reducing stress symptoms during middle age.

Our results are in line with evidence showing that working activity represents a key component of CR [55–58], as indicated by better cognitive abilities in adults with more complex jobs compared with those with less-engaging jobs [59]. Evaluating job-related information when assessing CR is therefore of primary importance since working in highly engaging and motivating jobs involves cognitive processes that in turn contribute to both CR and mental health. Critically, work-related activities stimulate mid-life cognitive engagement, and overall protect against the risk of dementia beyond education [58, 60]. It is still possible, however, that the advantage of more demanding jobs on reducing stress-related symptoms also reflects the intrinsic cognitive or emotional strength of individuals more capable to manage stress symptoms, besides their choice of more challenging jobs.

Our data also extend previous findings that linked working activity with cognition in healthy adults by including a measure of mental health integrity. This echoes recent data that found such a link in pathological adults [61]. Moreover, our work suggests the importance of including middle aged participants in studies focusing on the clinical psychology and neuropsychology of ageing. People aged between 40 and 60 years are often neglected in these studies, on the ground that more informative and larger changes are observable when comparing young to older adults [62, 63]. Indeed, most studies of the ageing brain are dominated by cross-sectional investigations typically comparing university students (about 20 years old) to pensioners (about 60–80 years old) [64], and therefore assuming a linear trajectory of change, with performance in midlife expected to fall midway between young and old age. Consequently, non-linear trajectories of age-related changes are rarely tested even when middle agers are part of the sample [65, 66].

On the contrary, middle adulthood is often a rich and active stage in most people’s lives [67, 68], with cognitive decline starting from mid-age, for instance in terms of impoverished processing speed, memory and executive functions [69–74]. These age-related behavioural changes parallel alterations in brain structure and function, especially in brain regions such as the prefrontal cortex, the medial temporal areas and the hippocampus. These areas show accelerated shrinkage [75–78], and white matter decreases in volume [79] or myelin content which starts in the fourth decade of life [34], pointing to the importance of studying middle age for signalling later age-related brain changes [80].

In conclusion, our data showed that CR protects mental health. Specifically, engaging and motivating professional activities (indexed by higher CRI-Working Activity) are linked to lower stress, especially in adults between the age of 40 and 60, i.e. middle agers. These results therefore extend past research indicating that CR attenuates the effect of age on cognitive decline by showing that such attenuation can also be related to mental health. Considering the complex profile of middle agers, future studies should further examine the elaborate dynamics between CR, cognitive mechanisms and neurodegenerative processes, also using a longitudinal approach. Such studies may also complement the current correlational approach with one looking at the possible causal role of CR and the type of professional activities on mental health, as well as the variability in cognitive and emotional profile of the sample. This would also allow us to more clearly define the directionality between higher CR and better mental health, which is currently not fully established. Finally, as all self-report measures, the CRI-q questionnaire relies on participants’ self-report and may therefore reflect poor or incomplete recall of information, which could be complemented by additional independent measures.

## Data Availability

Data and statistical analyses are available upon request.
